# Immediate *in vivo* target-specific cancer cell death after near infrared photoimmunotherapy

**DOI:** 10.1186/1471-2407-12-345

**Published:** 2012-08-08

**Authors:** Makoto Mitsunaga, Takahito Nakajima, Kohei Sano, Gabriela Kramer-Marek, Peter L Choyke, Hisataka Kobayashi

**Affiliations:** 1 Molecular Imaging Program, Center for Cancer Research, National Cancer Institute, National Institutes of Health, Building 10, Room B3B69, MSC1088, Bethesda, MD, 20892-1088, USA; 2 Radiation Oncology Branch, Center for Cancer Research, National Cancer Institute, National Institutes of Health, Bethesda, MD, 20892, USA

**Keywords:** Photoimmunotherapy, Theranostics, Cell death, Epidermal growth factor receptor, Molecular targeting, Monoclonal antibody, Bioluminescence imaging

## Abstract

**Background:**

Near infrared (NIR) photoimmunotherapy (PIT) is a new type of cancer treatment based on a monoclonal antibody (mAb)-NIR phthalocyanine dye, (IR700) conjugate. *In vitro* cancer-specific cell death occurs during NIR light exposure in cells previously incubated with mAb-IR700 conjugates. However, documenting rapid cell death *in vivo* is more difficult.

**Methods:**

A luciferase-transfected breast cancer cell (epidermal growth factor receptor+, MDA-MB-468luc cells) was produced and used for both *in vitro* and *in vivo* experiments for monitoring the cell killing effect of PIT. After validation of cytotoxicity with NIR exposure up to 8 J/cm^2^*in vitro*, we employed an orthotopic breast cancer model of bilateral MDA-MB-468luc tumors in female athymic mice, which subsequently received a panitumumab-IR700 conjugate *in vivo*. One side was used as a control, while the other was treated with NIR light of dose ranging from 50 to 150 J/cm^2^. Bioluminescence imaging (BLI) was performed before and after PIT.

**Results:**

Dose-dependent cell killing and regrowth was successfully monitored by the BLI signal *in vitro*. Although tumor sizes were unchanged, BLI signals decreased by >95% immediately after PIT *in vivo* when light intensity was high (>100 J/cm^2^), however, in mice receiving lower intensity NIR (50 J/cm^2^), tumors recurred with gradually increasing BLI signal.

**Conclusion:**

PIT induced massive cell death of targeted tumor cells immediately after exposure of NIR light that was demonstrated with BLI *in vivo*.

## Background

Conventional cancer therapies cause damage or toxicity in normal tissues, thus requiring dose reductions, which, in turn, limit the effectiveness of such agents [1,2]. In general, treatments that maximize target-cell killing while minimizing damage to normal cells are highly desirable. Targeted molecular cancer therapies offer the promise of more effective tumor targeting with fewer side effects than conventional cancer therapies, however, only limited success has thus far, been achieved. Combining drugs with activating physical energy, such as light or heat, is a potential method of improving therapeutic selectivity. We recently reported a new type of highly selective cancer therapy, termed “photoimmunotherapy” or PIT, which utilizes a monoclonal antibody (mAb)-bound to the photosensitizing phthalocyanine dye, IRDye700DX (IR700) to target cancer cells and an exposure of near infrared (NIR) light to specifically kill those cells. Remarkably, the mAb-IR700 conjugate is only active as a therapeutic agent, when it is bound to the target cell membrane; otherwise it had no effect on adjacent non-expressing cells [3]. Following NIR light exposure, immediate, target-selective necrotic cell death was observed *in vitro* using cytotoxicity assays, however, *in vivo* assessment of rapid cell death before decreasing tumor size is more challenging. Although progressive tumor shrinkage *in vivo* was observed 3-4 days after PIT, even after only a single administration of mAb-IR700 and a single exposure of NIR light, nonetheless there is uncertainty over how quickly cell death occurs [4]. Such information could be useful in optimizing PIT dosing and light exposure.

Bioluminescence (BLI) is a well established method of determining *in vivo* viability [5,6], since the BLI reaction requires both oxygen and ATP to actively transport the substrate luciferin and subsequently catalyze the photochemical reaction [7]. In this study we used BLI to monitor the kinetics of tumor cell death after PIT in epidermal growth factor receptor (EGFR) expressing orthotopic breast tumors after the mouse received anti-EGFR panitumumab-IR700 conjugate (Pan-IR700) followed by varying intensities of NIR light. Results were compared to identical tumors that were not exposed to NIR in the same mice. This method allows for the detection of massive cellular death *in vivo* immediately after PIT.

## Methods

### Reagents

A water soluble, silicon-phthalocyanine derivative, IRDye 700DX NHS ester (IR700; C_74_H_96_N_12_Na_4_O_27_S_6_Si_3_, molecular weight of 1954.22) was obtained from LI-COR Bioscience (Lincoln, NE). Panitumumab, a fully humanized IgG_2_ mAb directed against the human EGFR, was purchased from Amgen (Thousand Oaks, CA). All other chemicals were of reagent grade.

### Synthesis of IR700-conjugated panitumumab

Panitumumab (1 mg, 6.8 nmol) was incubated with IR700 (66.8 μg, 34.2 nmol, 5 mmol/L in DMSO) in 0.1 mol/L Na_2_HPO_4_ (pH 8.5) at room temperature for 2 h. The mixture was purified with a Sephadex G50 column (PD-10; GE Healthcare, Piscataway, NJ). The protein concentration was determined with Coomassie Plus protein assay kit (Thermo Fisher Scientific Inc, Rockford, IL) by measuring the absorption at 595 nm with spectroscopy (8453 Value System; Agilent Technologies, Santa Clara, CA). The concentration of IR700 was measured by absorption with spectroscopy to confirm the number of fluorophore molecules conjugated to each mAb molecule. The number of IR700 per antibody was ~3.

### Cells

EGFR-expressing MDA-MB-468luc, stable luciferase-transfected cells [8] were grown in RPMI 1640 supplemented with 10% fetal bovine serum and 1% penicillin/streptomycin in tissue culture flasks in a humidified incubator at 37°C in an atmosphere of 95% air and 5% carbon dioxide. Balb/3T3 cells (ATCC, Rockville, MD) were used as a control in the same culture condition.

### Fluorescence microscopy

To detect the antigen specific localization of IR700, fluorescence microscopy was performed (BX51 or IX81; Olympus America, Melville, NY). MDA-MB-468luc or 1:1 mixture of MDA-MB-468luc and Balb/3T3 cells were seeded on a cover glass-bottomed dishes and incubated 24 h. Pan-IR700 was added to the culture medium at 10 μg/mL and incubated for 6 h at 37°C, then cells were washed with PBS. The filter was set to detect IR700 fluorescence with a 590–650 nm excitation filter, and a 665–740 nm band pass emission filter.

### *In vitro* PIT

Cells were seeded into 96 well plate or 35 mm cell culture dishes and incubated 8 h. Medium was replaced with fresh culture medium containing 10 μg/ml of Pan-IR700 and incubated over night at 37°C. After washing with PBS, phenol red free culture medium was added. Then, cells were irradiated with a red light-emitting diode (LED), which emits light at 670 to 710 nm wavelength (L690-66-60; Marubeni America Co., Santa Clara, CA), and a power density of 25 mW/cm^2^ as measured with optical power meter (PM 100, Thorlabs, Newton, NJ).

### Phototoxicity assay

Cytotoxic effects of PIT with Pan-IR700 were determined with luciferase activity assay and flowcytometric LIVE⁄DEAD® Fixable Green Dead Cell Stain Kit (Invitrogen, Carlsbad, CA), which can detect compromised cell membranes. For luciferase activity assay, D-luciferin (Gold Biotechnology, St. Louis, MO) was added to culture media at 150 μg/ml and analyzed on a bioluminescence imaging system (Photon Imager; Biospace Lab, Paris, France). For the flowcytometric assay, cells were trypsinized after treatment and washed with PBS. Green fluorescent reactive dye was added in the cell suspension and incubated at room temperature for 30 min, followed by analysis on a flow cytometer (FACS Calibur, BD Biosciences, San Jose, CA).

### Orthotopic breast tumor model

All *in vivo* procedures were conducted in compliance with the Guide for the Care and Use of Laboratory Animal Resources (1996), US National Research Council, and approved by the National Cancer Institute Animal Care and Use Committee. Six- to eight-week-old female homozygote athymic nude mice were purchased from Charles River (NCI-Frederick, Frederick, MD). During the procedure, mice were anesthetized with isoflurane. Two million MDA-MB-468luc cells were implanted into the mammary fat pads bilaterally. D-luciferin (15 mg/ml, 200 μl) was injected intraperitoneally into mice 6 days after cell implantation, and analyzed with Photon Imager for luciferase activity. Mice were selected for further study if their tumors demonstrated symmetry based on size and BLI signal.

### *In vivo* PIT with Pan-IR700

As there was no treatment effect for MDA-MB-468luc tumors after the single administration of unconjugated panitumumab, selected mice were randomized into 5 groups of 5 animals per group for the following treatments: (1) no treatment; (2) 100 μg of Pan-IR700 i.v., no NIR light exposure; (3) 100 μg of Pan-IR700 i.v., NIR light was administered at 50 J/cm^2^ on day 1 after injection; (4) 100 μg of Pan-IR700 i.v., NIR light was administered at 100 J/cm^2^ on day 1 after injection; (5) 100 μg of Pan-IR700 i.v., NIR light was administered at 150 J/cm^2^ on day 1 after injection. NIR light exposure was performed 8 days after cell implantation. Mice images were acquired over time with a fluorescence imager (Pearl Imager; LI-COR Biosciences) for detecting IR700 fluorescence, and Photon Imager for BLI. For analyzing fluorescence and BLI, regions of interest (ROI) of similar size were placed over the entire tumor. The average fluorescence intensity of each ROI was measured. When comparing fluorescence target-to-background ratios (TBR), ROIs were placed in the surrounding non-tumor region.

### Histological analysis

To evaluate serial histological changes immediately after PIT with various NIR light doses, microscopic study was performed (BX51, Olympus America). MDA-MB-468luc tumors were harvested in 10% formalin immediately after 50, 100, and 150 J/cm^2^ of NIR light exposure. Serial 10-μm slice sections were fixed on 2 glass slides with Hematoxylin and Eosin (H-E) staining.

### Statistical analysis

Data are expressed as means ± s.e.m. from a minimum of three experiments. Statistical analyses were carried out using a statistics program (GraphPad Prism; GraphPad Software, La Jolla, CA). Student’s *t* test was used to compare the treatment effects with that of controls.

## Results

### Target-specific, dose-dependent NIR light-induced necrotic cell death in response to Pan-IR700 mediated PIT

**Figure 1:**
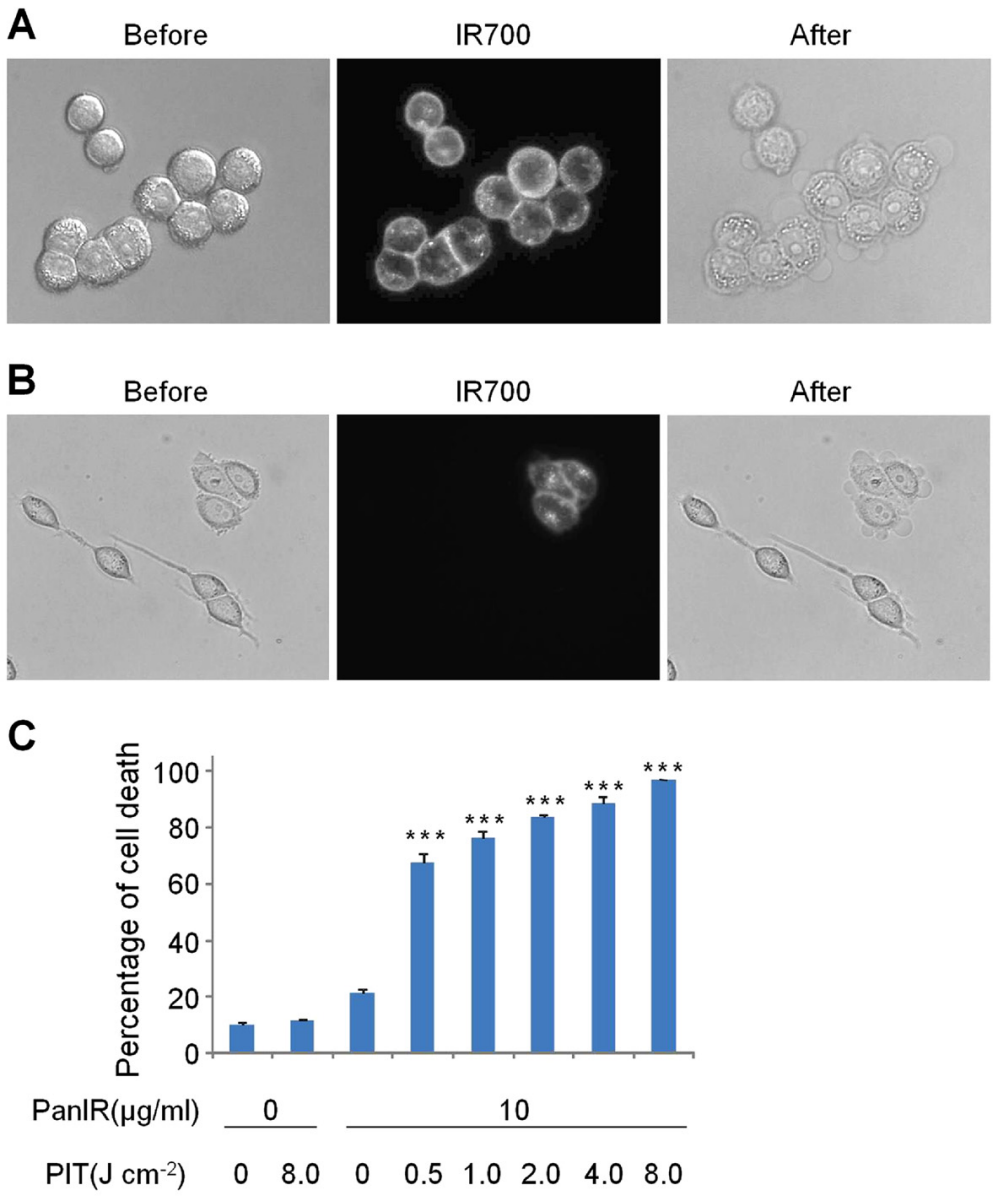
**EGFR-specific PIT *in vitro.*** (**A**) MDA-MB-468luc cells were treated with Pan-IR700 and observed with microscopy (Before and after Pan-IR700). Necrotic cell death was observed upon excitation with NIR light (After). (**B**) Both EGFR positive (MDA-MB-468luc) and negative (Balb/3T3) cells were co-cultured and treated with Pan-IR700 (Before and after Pan-IR700). EGFR-specific necrotic cell death was observed (After). (**C**) NIR light irradiation results in dose dependent cell death as determined by the cytotoxicity assay. (*n* = 3, *** *P*<0.001 vs. non treatment control, Student’s *t* test).

Fluorescence microscopy was performed to confirm target-specific localization of Pan-IR700. IR700 was mainly localized to the cell membrane and lysosomes of EGFR positive MDA-MB-468luc cells. When these cells were observed during continuous NIR light exposure, almost immediate swelling, budding and rupture of the lysosome was observed leading to irreversible cell death (Figure 1A). MDA-MB-468luc and EGFR negative Balb/3T3 cells were co-incubated to confirm cell-specific killing. Pan-IR700 did not localize to Balb/3T3 cells which were not killed by exposure to NIR (Figure 1B). To confirm that rapid phototoxic cell death had occurred, we used the LIVE/DEAD assay, which can detect early cell membrane damage. Cell death increased significantly in a dose dependent manner with the intensity of NIR light (*P*<0.001) but no cell death was observed in the absence of Pan-IR700 incubation or in the absence of light (Figure 1C).

### Bioluminescence imaging of rapid tumor killing in response to Pan-IR700 mediated PIT

**Figure 2:**
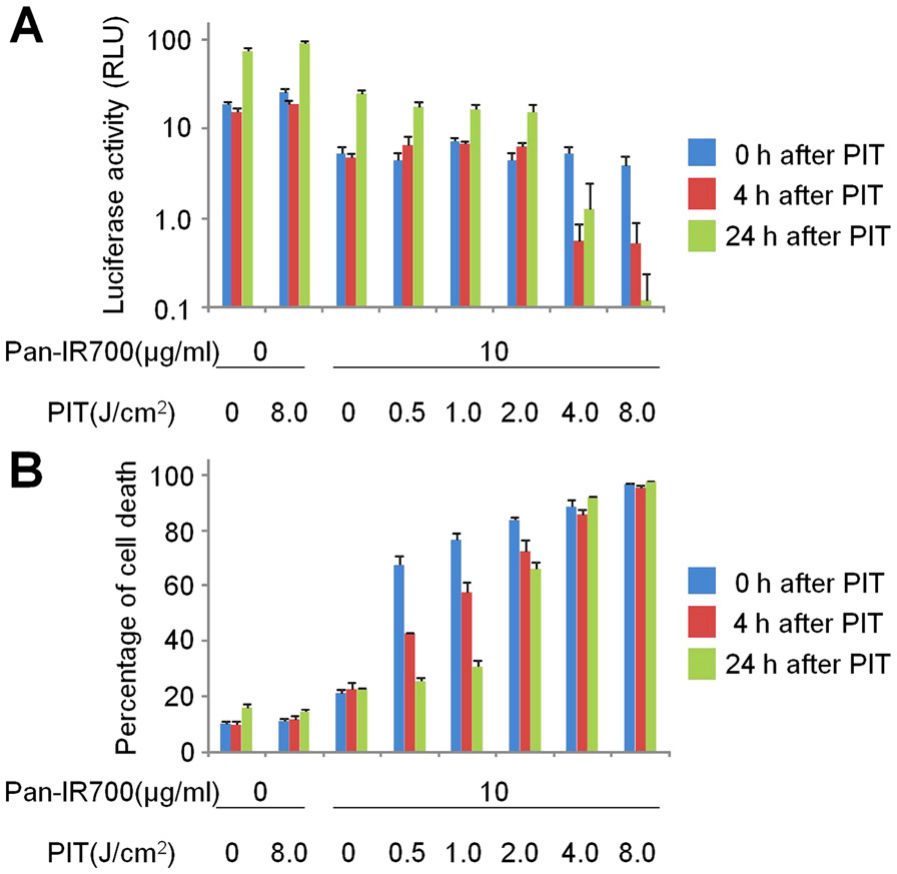
** Effect of phototoxicity in response to Pan-IR700 mediated PIT.** Cell viability was analyzed with (**A**) bioluminescence and (**B**) fluorescence LIVE/DEAD cytotoxicity assay after NIR light irradiation in MDA-MB-468luc cells.

We next examined the effect of rapid Pan-IR700-mediated phototoxicity using BLI *in vitro*. Viable MDA-MB-468luc cells ranging from 3.1 × 10^4^ to 1.0 × 10^6^ were detected with BLI prior to NIR exposure (Additional file 1 Figure S1). Low levels of NIR light irradiation dose (~2.0 J/cm^2^) resulted in initially decreased BLI followed by recovery of signal as the cells regrew. In contrast, higher levels of NIR light exposure (8.0 J/cm^2^) resulted in permanent loss of BLI signal. Interestingly, BLI activity did not immediately disappear after NIR light irradiation even with high levels of exposure (Figure 2A) probably because the budding cells released all the necessary components of the BLI photochemical reaction (luciferase, ATP, oxygen, and luciferin) into the surrounding well permitting the reaction to persist even while the cells were evidently dead based on the cytotoxicity assay, which showed prompt increases in the number of dead cells. Exposure to higher doses of NIR led to the death of more than 90% of the cells and BLI signal remained reduced over time (Figure 2B).

### *In vivo* monitoring of the acute therapeutic effects of Pan-IR700 mediated PIT

**Figure 3:**
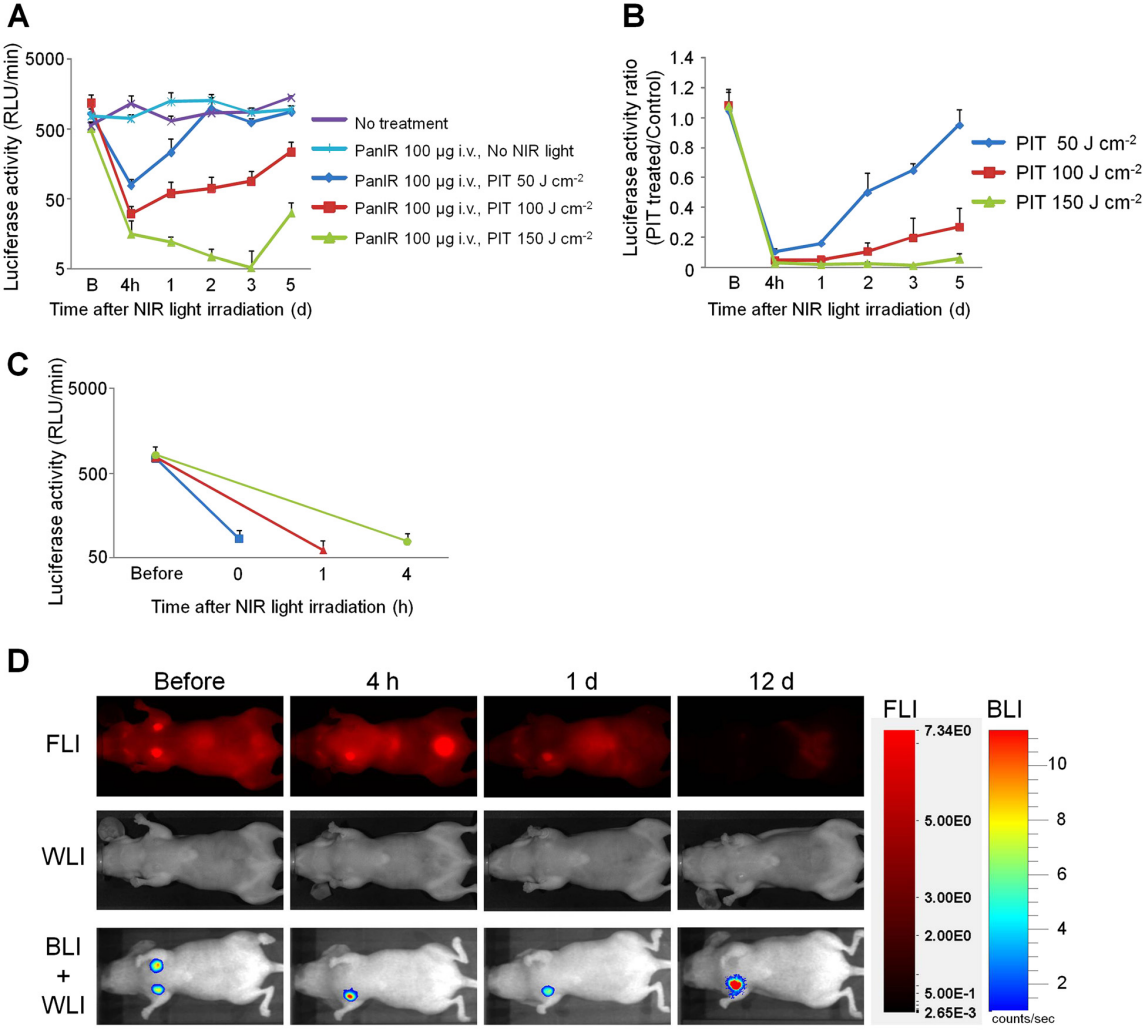
*** In vivo *fluorescence and bioluminescence imaging of orthotopic breast tumors in response to Pan-IR700 mediated PIT.** (**A**) Monitoring bioluminescence activity in response to Pan-IR700 mediated PIT (*n* = 5 each). B: just before NIR light treatment. Bioluminescence signals of tumors in 100 and 150 J/cm^2^ treated groups showed significant difference with that in control or 50 J/cm^2^ treated group at all time points examined after PIT (*P*<0.001). (**B**) Monitoring bioluminescence activity ratio of treated tumor to internal control (*n* = 5 each). B: just before NIR light treatment. Bioluminescence signals of tumors in 100 and 150 J/cm^2^ treated groups showed significant difference with that in control or 50 J/cm^2^ treated group at all time points examined after PIT (*P*<0.001). (**C**) Rapid changes of bioluminescence activity in response to Pan-IR700 mediated PIT (*n* = 5 each). Before: 1 d before NIR light treatment. (**D**) IR700 fluorescence and bioluminescence imaging in response to Pan-IR700 mediated PIT. Representative images are shown. Before: just before NIR light treatment, FLI: IR700 fluorescence imaging, WLI: white light imaging, BLI: bioluminescence imaging.

Orthotopically implanted MDA-MB-468luc breast cancer cells were injected into mammary fat pads bilaterally. Tumors were first recognized at approximately 4 mm in diameter and 6 days after cell injection. BLI was performed at 6 days to select 25 mice (5 groups of 5 mice per group) with symmetrical levels of bioluminescence. In addition to symmetry on BLI we also selected for tumors of similar size and those that demonstrated similar IR700 fluorescence at day 7 post cell injection. Mice received Pan-IR700 at day 6 and typically the right sided tumors were exposed to NIR light irradiation at day 7, while the left sided tumors were covered. BLI was obtained at 4 h intervals since this is how long it took for the BLI signal to extinguish after luciferin administration. BLI showed a significant decrease in signal compared to baseline within 4 h after NIR light exposure, compared with the various control groups: Pan-IR700+ NIR- and Pan-IR700-, NIR + and Pan-IR700-, NIR-. BLI revealed that irradiating with 50 J/cm^2^ resulted in the recovery of BLI signal over time, whereas tumors irradiated with 150 J/cm^2^ did not show recovery (Figure 3B). In order to demonstrate immediate cell killing, we randomized 15 mice in 3 groups 24 h before NIR irradiation, and irradiated each tumor with 100 J/cm^2^, followed by BLI at 0, 1 and 4 h after irradiation. As shown in Figure 3C, BLI signal decreased immediately after NIR light irradiation probably because ATP is hydrolyzed during PIT preventing bioluminescence reactions. Since IR700 is also a fluorescent dye, we were also able to observe PIT effects with a fluorescence camera. IR700 fluorescence was detected in MDA-MB-468luc tumors before NIR light irradiation with a high target-to-background ratio (TBR = ~ 8), but was markedly decreased to nearly background level (TBR = ~1) immediately after NIR light irradiation. In contrast, IR700 fluorescence intensity in non-treated (Pan-IR700+, NIR-) control tumors decreased slowly with time commensurate with the conjugate’s pharmacokinetics (Figure 3D).

### Histological analysis

**Figure 4:**
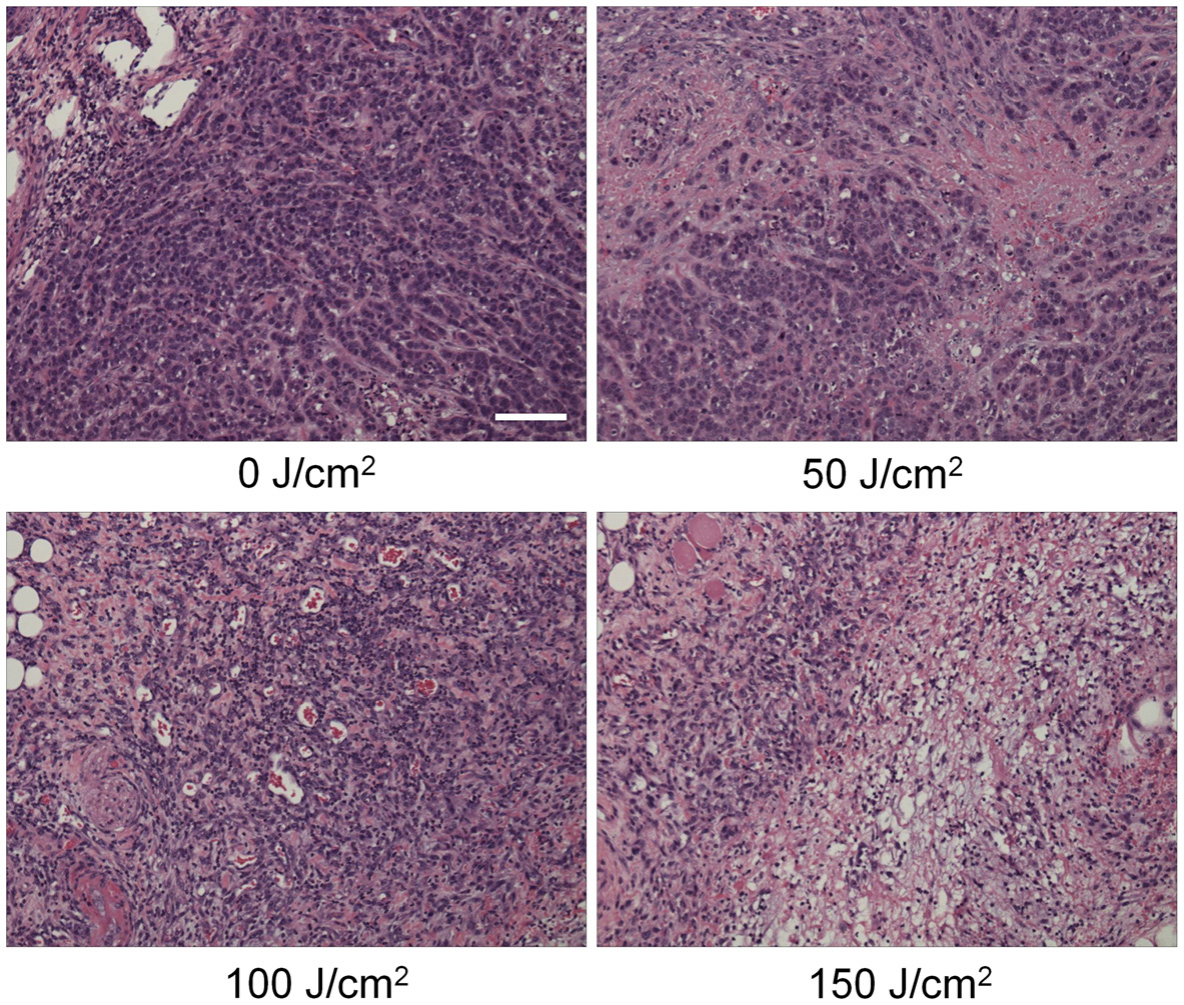
** Histological findings immediately after PIT with various NIR exposures.** Histological specimens of MDA-MB-468luc tumors, which were treated with PIT at 0, 50, 100, and 150 J/cm^2^, are shown. All specimens are stained with Hematoxylin and Eosin. A few scattered clusters of damaged tumor cells are seen within a background of diffuse cellular necrosis and micro-hemorrhage immediately after PIT with 50 J/cm^2^. Necrotic damage was more intense when higher intensity NIR light was administered. Scale indicates 50μm.

Microscopic evaluation of treated tumors revealed diffuse necrosis and microhemorrhage with scattered clusters of damaged tumor cells after PIT with 50-150 J/cm^2^. Necrotic damage was more intense and fewer tumor cells remained, when higher energy (150 J/cm^2^) NIR light was administered (Figure 4).

## Discussion

This study demonstrates that target-selective accumulation of Pan-IR700 in MDA-MB-468luc tumors resulted in rapid cell death, which was dose dependent based on the NIR light intensity in the range of 50-150 J/cm^2^. Immediate cell death after exposure to NIR light could be validated by BLI. Bioluminescence signals decreased to less than 3% just after PIT treatment with 100 J/cm^2^ of NIR light irradiation, indicating near instantaneous tumor cell killing *in vivo*. Fluorescence signal also decreased immediately after NIR exposure of PIT as shown in Figure 3D. However, this immediate decrease of IR700 fluorescence could be induced partly by photo-bleaching of IR700. This immediate cell death suggests that the mechanism of PIT-induced tumor cell death is necrosis via direct physical injury such as pressure waves induced by local heat elevation and not through slower death pathways such as apoptosis or autophagy. Lower doses of light resulted in incomplete cell killing causing tumor regrowth as demonstrated by increasing BLI signal. Higher doses of light (e.g. 150 J/cm^2^) resulted in complete responses. Thus, BLI are able to monitor therapeutic responses to PIT.

Paradoxically, the BLI results appeared to be less rapid when cells were tested *in vitro.* Although Pan-IR700 treated MDA-MB-468luc cells were rapidly and selectively killed in response to NIR light irradiation, BLI appeared to show that cell killing was slower than *in vivo* cell killing [3]. Nearly instantaneous cell killing was demonstrated with the LIVE/DEAD cytotoxicity assay, which detected early cellular membrane damage after low levels (less than 2 J/cm^2^ ) of NIR light while BLI signal was reduced only after 4 h post NIR exposure [9,10]. These data suggest that disrupted cellular membranes, which can be defined as “dead” by LIVE/DEAD staining assay may undergo rapid cell surface repair to reseal cellular membrane, while, aggressively disrupted cells after strong NIR irradiation (more than 2 J/cm^2^) were irreversibly damaged and could not repair the disrupted membrane [11]. However, an additional factor is that BLI signal was artifactually preserved *in vitro*. Even after severe mechanical disruption of a cell membrane all the necessary elements for BLI including ATP, oxygen and luciferin still exist within the well in sufficient concentrations to produce a photochemical reaction. In contrast, when PIT is performed *in vivo*, released ATP is rapidly hydrolyzed in the local microenvironment resulting in rapid loss of BLI signal. Thus, BLI may be a more valuable tool for *in vivo* monitoring than for *in vitro* monitoring of cell therapies, which are based on rapid physical damage, as opposed to chemical or biological damage to cancer cells.

Fluorescent proteins (FPs) are a potential alternative for monitoring tumor growth *in vivo*[12-15]. Fluorescence imaging using FPs are better direct and stable method for longitudinal monitoring therapeutic effects of photo-therapy [16,17] for days or weeks than the bioluminescence imaging, which is used in this study, because most of FPs are stable in solution for days *in vitro*[18] and fluoresced before FPs are taken up and catabolized by macrophages *in vivo*[12]. Therefore, fluorescence imaging has already been used for longitudinal monitoring of therapeutic effects of PIT [4]. However, PIT-induced immediate massive cell death, which rarely happens in cancer therapy, did not depict after with the fluorescence imaging for hours but depicted with the bioluminescence imaging because fluorescent substances are stable than ATP, which hydrized immediately *in vivo*. Therefore, the bioluminescence imaging is theoretically and practically the appropriate method for detecting this unique PIT-induced immediate massive cell death.

Proper controls are vital to prove that the cell killing is related to the combination of Pan-IR700 and NIR light exposure. We achieved this by implanting breast tumors bilaterally in the fat pads of mice and selecting for mice with tumors that were symmetric in size and BLI/fluorescence signals just before PIT. Controls included tumors that did not receive Pan-IR700 but did receive light, tumors that received Pan-IR700 but did not receive NIR light and those that received neither agent nor light. No cell killing was observed in these controls. In contrast to a previous study of PIT, which employed a subcutaneous xenograft, we employed an orthotopic bilateral breast cancer tumor model and used one tumor as an internal control [19]. Such symmetry is more easily achieved in orthotopic vs. subcutaneous models and this model was also able to demonstrate that response was dose dependent with regard to light exposure.

## Conclusions

Immediate cytotoxicity induced by PIT was demonstrated using bioluminescence imaging *in vivo*. The immediate cell killing demonstrated by BLI strongly suggests that the mechanism of action of PIT is necrosis due to rapid mechanical membrane disruption caused by local heating and induced pressure waves. This is supported by direct observational microscopic evidence that rapid cell swelling and budding is seen in cells previously treated with a mAb-IR700 conjugate and subsequently exposed to NIR light. This data support the concept that PIT could be highly controlled by appropriate dosing of light to specific tumor cells identified by their IR700 fluorescence, resulting in a true “see and treat” paradigm that could be useful during surgical or endoscopic procedures. Practically, surgeons or endoscopy physicians could “see” tumors with the fluorescence of IR700, and then “treat” them by surgery combined with exposing the NIR light to achieve complete treatment of a patient. This result suggests that physicians should not misread remaining persistent fluorescence signal as a sign of survived tumors.

## Abbreviations

NIR: Near infrared; PIT: Photoimmunotherapy; mAb: Monoclonal antibody; EGFR: Epidermal growth factor receptor; BLI: Bioluminescence imaging; Pan: Panitumumab; IR700: IRDye700DX; ROI: Regions of interest; TBR: Target-to-background ratios.

## Competing interests

The authors declare that they have no competing interests.

## Authors’ contributions

M.M. conducted experiments, performed analysis and wrote the manuscript; T.N., K.S. and G.K. conducted experiments and performed analysis; P.L.C. wrote the manuscript and supervised the project; and H.K. planned and initiated the project, designed and conducted experiments, wrote the manuscript, and supervised the entire project. All authors read and approved the final manuscript.

## Pre-publication history

The pre-publication history for this paper can be accessed here:

http://www.biomedcentral.com/1471-2407/12/345/prepub

## Supplementary Material

Additional file 1 Figure S1 Sensitivity of bioluminescence assay in serially diluted viable MDA-MB-468luc cells. BLI signal could be detected in as few as 3.1x10^4^ cells MDA-MB-468luc. Click here for file
