# Supplementary figures and images for: Immediate in vivo target-specific cancer cell death after near infrared photoimmunotherapy
Source: BMC Cancer. 2012 Aug 8;12:345. doi: 10.1186/1471-2407-12-345 (PMC3502522; doi:10.1186/1471-2407-12-345)

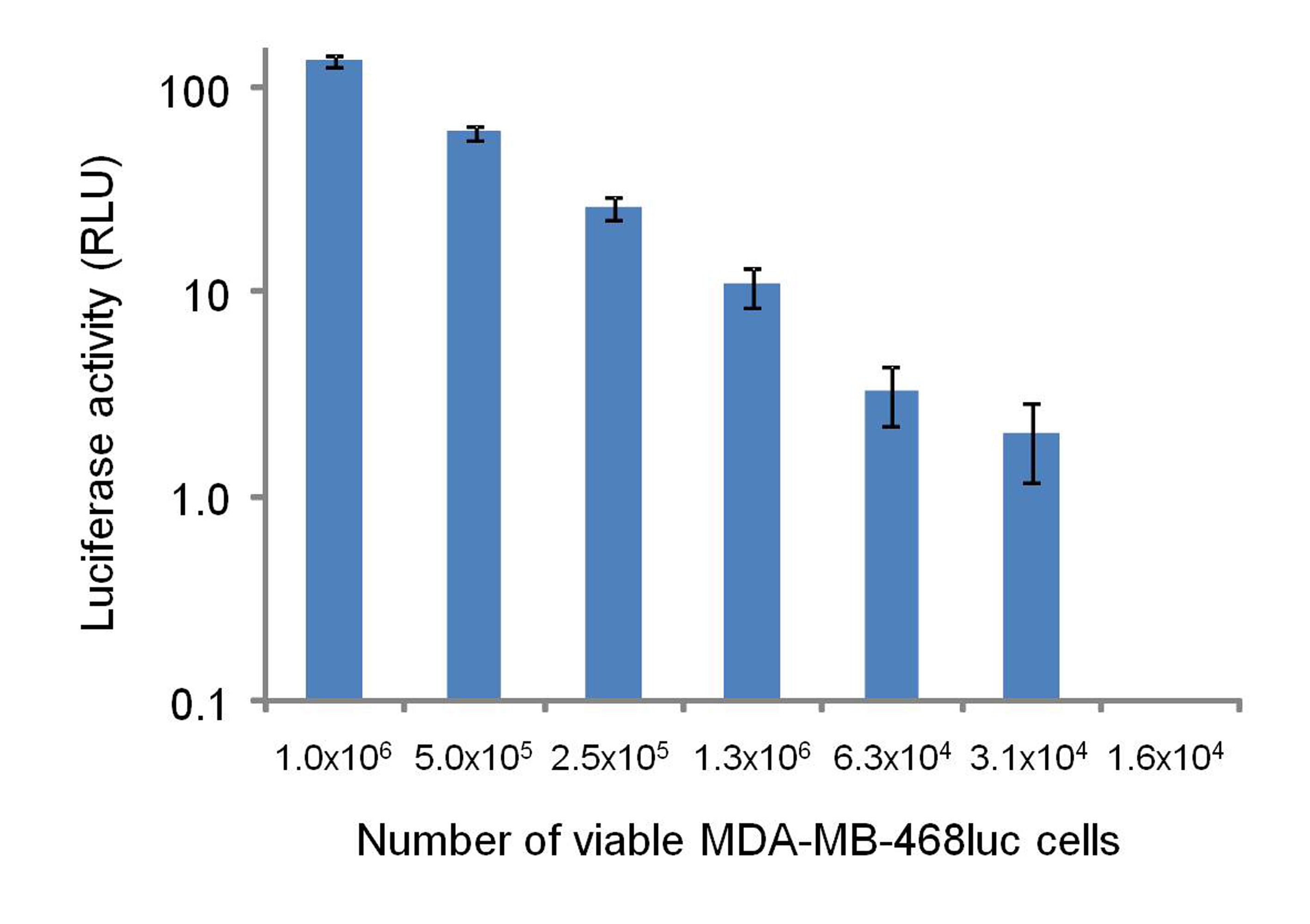

Supplement: Additional file 1 — Figure S1 Sensitivity of bioluminescence assay in serially diluted viable MDA-MB-468luc cells. BLI signal could be detected in as few as 3.1x10^4^ cells MDA-MB-468luc. [file 1471-2407-12-345-S1.jpeg]
